# Induction of controlled hypoxic pregnancy in large mammalian species

**DOI:** 10.14814/phy2.12614

**Published:** 2015-12-10

**Authors:** Kirsty L. Brain, Beth J. Allison, Youguo Niu, Christine M. Cross, Nozomi Itani, Andrew D. Kane, Emilio A. Herrera, Dino A. Giussani

**Affiliations:** ^1^ Department of Physiology, Development and NeuroscienceUniversity of CambridgeCambridgeUK; ^2^ Laboratorio de Fisiología y Fisiopatología del DesarrolloInstituto de Ciencias Biomédicas (ICBM)Facultad de MedicinaUniversidad de ChileSantiagoChile

**Keywords:** Cardiovascular, chronic hypoxia, fetus, pregnancy

## Abstract

Progress in the study of pregnancy complicated by chronic hypoxia in large mammals has been held back by the inability to measure long‐term significant reductions in fetal oxygenation at values similar to those measured in human pregnancy complicated by fetal growth restriction. Here, we introduce a technique for physiological research able to maintain chronically instrumented maternal and fetal sheep for prolonged periods of gestation under significant and controlled isolated chronic hypoxia beyond levels that can be achieved by habitable high altitude. This model of chronic hypoxia permits measurement of materno‐fetal blood gases as the challenge is actually occurring. Chronic hypoxia of this magnitude and duration using this model recapitulates the significant asymmetric growth restriction, the pronounced cardiomyopathy, and the loss of endothelial function measured in offspring of high‐risk pregnancy in humans, opening a new window of therapeutic research.

## Introduction

Fetal hypoxia is one of the most common problems associated with high‐risk pregnancy (Giussani and Davidge 2013; Giussani 2015). Acute episodes of fetal hypoxia can occur during complicated birth, for instance as a result of compressions of the umbilical cord during prolonged labor (Huch et al. 1977). In contrast to the adult individual that is able to trigger cardioventilatory responses to reduced oxygenation, the fetal defense to acute hypoxia is contingent on the fetal cardiovascular system. The sheep fetus has long been the experimental model of choice for investigating fetal cardiovascular function in vivo. Using this model, the effects of short‐term episodes of hypoxia on fetal cardiovascular function have been studied extensively. In response to acute hypoxia, the fetal heart rate falls and there is a redistribution of blood flow away from peripheral circulations toward essential vascular beds, such as those perfusing the brain – the so‐called fetal brain sparing effect (Rudolph 1984; Giussani 2015). The physiology underlying the fetal cardiovascular responses to acute hypoxia is also well delineated and includes carotid chemoreflex activation, neuroendocrine responses, and the contribution of a local vascular oxidant tone determined by the interaction between nitric oxide and reactive oxygen species (Giussani et al. 1994; Giussani 2015).

In addition to high‐altitude pregnancy (Soria et al. 2013) and maternal smoking (Longo 1976), chronic fetal hypoxia is also associated with increased placental vascular resistance, as can occur during pregnancy complicated by preeclampsia, placental insufficiency, or placental infection (see Giussani and Davidge 2013; Giussani 2015). In marked contrast to knowledge of the fetal physiological compensatory responses to acute hypoxia, comparatively little is known about the effects on fetal cardiovascular function of long‐term hypoxic pregnancy. Progress in this field has been hampered in part by the inability to induce controlled reductions in oxygenation which are continuously monitored for prolonged periods of time during pregnancy in large mammalian species, such as sheep. Relative to humans, rodents are born immature and the development of the cardiovascular system continues into the postnatal period (Sissman 1970; Monie 1976). Therefore, the relative effects on mammalian fetal cardiovascular function of prematurity and of chronic fetal hypoxia in studies involving hypoxic pregnancy in mice and rats are unclear (Jang et al. 2015). Seminal studies using pregnant sheep have induced chronic fetal hypoxia by impairing utero‐placental perfusion either by compression of the uterine or umbilical arteries, by placental embolization, by placental restriction through carunclectomy, or by exposure of the pregnancy to high ambient temperature (see Barry et al. 2008; Giussani 2015). All such methods reduce nutrient as well as oxygen delivery to the fetus. Therefore, the partial contributions to alterations in fetal growth and physiology of fetal undernutrition versus fetal hypoxia under these conditions also remain unclear. A series of elegant studies have exploited the natural hypobaric hypoxia of life at high altitude to investigate the effects of chronic hypoxic pregnancy on fetal cardiovascular function (e.g., Kamitomo et al. 1993; Kamitomo et al. 1992). While these studies have pushed the field forward significantly, exposure of pregnant ewes to altitudes between 3000 and 4000 m above sea level does not induce fetal growth restriction (FGR) consistently, as the reductions in fetal oxygenation achieved are milder than those measured in human FGR pregnancy (Hecher et al. 1995). This article introduces to the field a new technique able to measure controlled significant reductions in oxygenation, akin to those measured in human FGR pregnancy, over prolonged periods of time in the sheep. The application of the new method is validated by investigating the effects of significant chronic hypoxia during the last third of pregnancy in sheep on fetal growth as well as fetal cardiac and endothelial function.

## Methods

### Surgical preparation

All procedures were performed under the UK Animals (Scientific Procedures) Act 1986 and were approved by the Ethical Review Committee of the University of Cambridge. At 100 ± 1 days gestational age (term *ca*. 145 days), pregnant Welsh mountain ewes carrying singleton pregnancies determined by ultrasound scan (Toshiba Medical Systems Europe, Zoetermeer, the Netherlands) underwent a laparotomy under general anesthesia. In brief, food but not water was withdrawn for 24 h prior to surgery. Anesthesia was induced by Alfaxan (1.5–2.5 mg kg^−1^ i.v. alfaxalone; Jurox Ltd., Worcestershire, UK) and general anesthesia (1.5–2.0% isofluorane in 60:40 O_2_:N_2_O) maintained by use of a positive pressure ventilator (Datex‐Ohmeda Ltd., Hatfield, Hertfordshire, UK). Antibiotics (30 mg kg^−1^ i.m. procaine benzylpenicillin; Depocillin; Intervet UK Ltd., Milton Keynes, UK) and an analgesic (1.4 mg kg^−1^ s.c. carprofen; Rimadyl; Pfizer Ltd., Kent, UK) were administered immediately before the start of surgery. Following a midline abdominal incision and uterotomy, the fetal hind limbs were exposed and the fetal sex was determined. If male, then the fetuses were chosen for this study. Female fetuses were used for another experiment. The fetus was returned into the intrauterine cavity, and the uterine and maternal abdominal incisions were closed in layers. A Teflon catheter (i.d. 1.0 mm, o.d. 1.6 mm, Altec, UK) was then placed in the maternal femoral artery and extended to the descending aorta, in addition to a venous catheter extended into the maternal inferior vena cava (i.d. 0.86 mm, o.d. 1.52 mm, Critchly Electrical Products, NSW, Australia). Catheters were filled with heparinized saline (80 I.U mL^−1^ heparin in 0.9% NaCl), tunneled subcutaneously, and exteriorized via a keyhole incision made in the maternal flank to be kept inside a plastic pouch sewn onto the maternal skin. Inhalation anesthesia was withdrawn and the ewe was ventilated until respiratory movements were observed. The ewe was extubated when spontaneous breathing returned and moved into a recovery pen adjacent to other sheep with free access to food and water. A total of 18 Welsh Mountain ewes carrying male singleton fetuses were surgically instrumented for this component of the study.

### Postoperative care

Following surgery, ewes were housed in individual floor pens with a 12 h:12 h light:dark cycle and free access to hay and water. Antibiotics (30 mg kg^−1^ i.m. procaine benzylpenicillin; Depocillin; Intervet UK Ltd., Milton Keynes, UK) were administered daily to the ewe for 5 days following surgery. From 103 days of gestation, ewes were fed daily a bespoke maintenance diet made up of concentrate and hay pellets to facilitate the monitoring of food intake (Cambridge ewe diet: 40 g nuts kg^−1^ and 3 g hay kg^−1^; Manor Farm Feeds Ltd.; Oakham, Leicestershire, UK). Generally, normal feeding patterns were restored within 24–48 h of recovery. On day 103 of gestation, ewes were randomly assigned to either of two experimental groups: normoxia (N: *n* = 10) or chronic hypoxia (H: *n* = 8).

**Figure 1:**
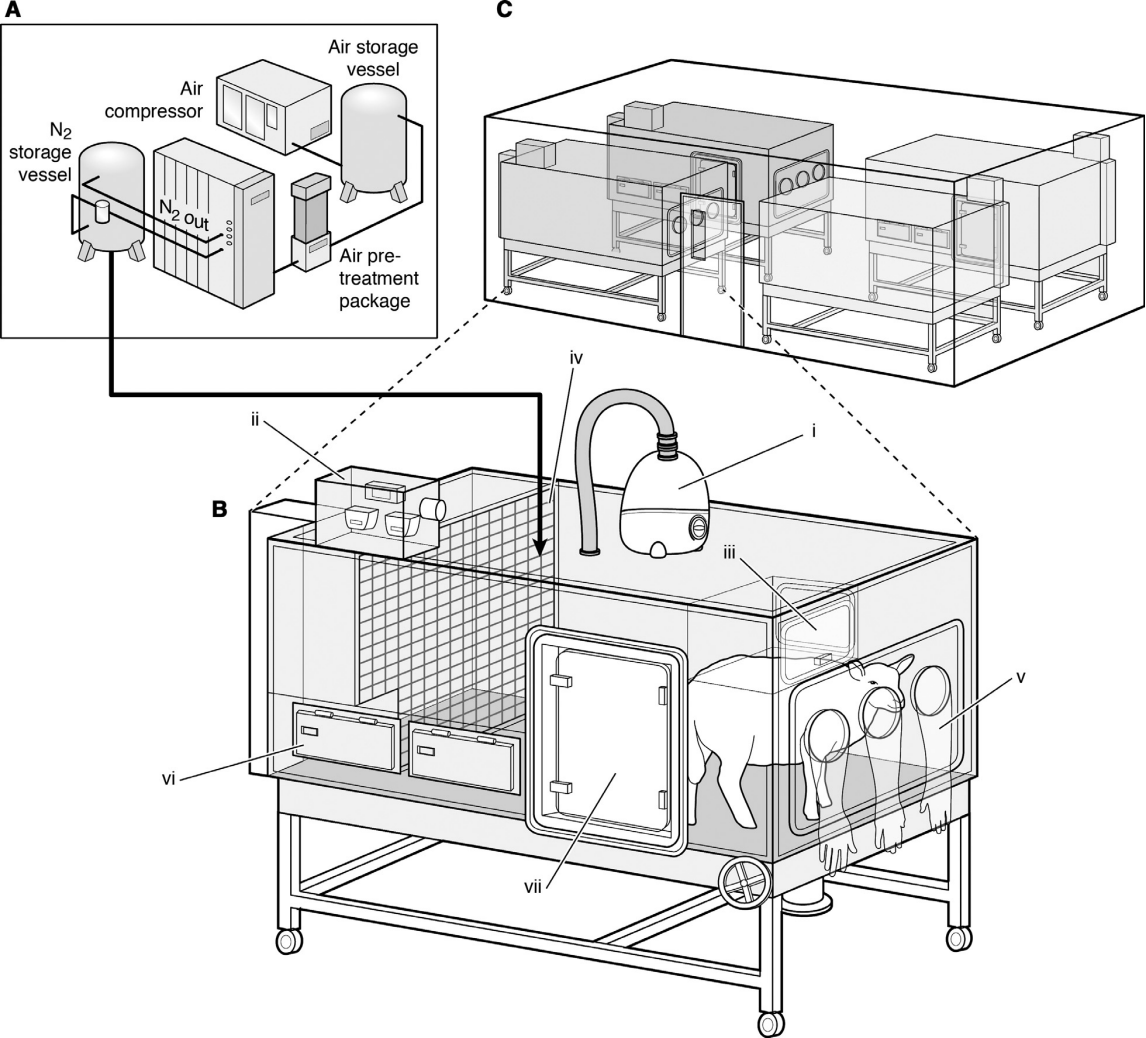
Isobaric hypoxic chambers and nitrogen‐generating system. A specially designed nitrogen‐generating system (A) supplied variable amounts of compressed air and nitrogen to four bespoke isobaric hypoxic chambers housed in the hypoxic chamber laboratory (B and C). Each chamber was equipped with an electronic servo‐controlled humidity cool steam injection system to return the appropriate humidity to the inspirate (i). Ambient po_2_, pco_2_, humidity, and temperature within each chamber were monitored via sensors (ii). For experimental procedures, each chamber had a double transfer port (iii) to internalize material and a manually operated sliding panel (iv) to bring the ewe into a position where daily sampling of blood could be achieved through glove compartments (v). Each chamber incorporated a drinking bowl on continuous water supply and a rotating food compartment (vi) for determining food intake. A sealed transfer isolation cart could be attached to a side exit (vii) to couple chambers together for cleaning.

Ewes allocated to chronic hypoxic pregnancy were housed in one of four bespoke isobaric hypoxic chambers (Telstar Ace, Dewsbury, West Yorkshire, UK; Fig. 1). These chambers were supplied with variable amounts of nitrogen and air provided via nitrogen generators and air compressors, respectively, from a custom‐designed nitrogen‐generating system (Domnick Hunter Gas Generation, Gateshead, Tyne & Wear, UK). The system operated continuously, automatically switching between adsorption beds of two nitrogen generators (Domnick Hunter N2MAX112 × 2) to ensure a constant provision of pure nitrogen gas. The purity of the nitrogen was monitored to ensure only gas of the required purity reached the application. Compressed air and compressed nitrogen were then piped to the laboratory containing the hypoxic chambers and gases were blended to requirements. The inspirate air mixture underwent a minimum of 12 changes per hour in each chamber and the incoming air mixture was passed via silencers able to reduce noise levels within the hypoxic chamber laboratory (76 dB(A)) and inside each chamber (63 dB(A)) to values lower than those necessary to abide by the Control of Noise at Work Regulations. This not only complied with human health and safety and animal welfare regulations, but also provided a tranquil environment for the animal inside each chamber. All chambers were equipped with an electronic automatic humidity cool steam injection system (1100‐03239 HS‐SINF Masalles, Barcelona, Spain) to ensure appropriate humidity in the inspirate (55 ± 10%). Ambient po_2_, pco_2_, humidity, and temperature within each chamber were monitored via sensors, displayed, and values recorded continuously via the Trends Building Management System of the University of Cambridge through a secure Redcare intranet. In this way, the percentage of oxygen in the isolators could be controlled with precision continuously over long periods of time. For experimental procedures, each chamber had a double transfer port to internalize material and a manually operated sliding panel to encourage the ewe into a position where daily sampling of blood could be achieved through glove compartments (Fig. 1). Each chamber incorporated a drinking bowl on continuous water supply and a rotating food compartment which could be removed for determining food intake. The chambers were transparent, allowing ewes to visualize each other. A transfer isolation cart could couple two chambers together, permitting ewes to move transiently to an adjacent chamber maintained at the same oxygen environment. This was necessary for cleaning the chambers, which occurred once per week. Therefore, all experimental and maintenance procedures could be carried out without interruption of the hypoxic exposure. Pregnancies assigned to the chronic hypoxia group were placed inside the chambers at 103 days of gestation under normoxic conditions (11 L sec^−1^ air, equating to 39.6 m^3^ h^−1^). At 105 days, pregnancies were exposed to approximately 10% O_2_ by altering the incoming inspirate mixture to 5 L sec^−1^ air: 6 L sec^−1^ N_2_.

### Blood sampling regimen and analysis

Samples of descending aortic maternal blood (0.3 mL) were taken daily for the measurement of maternal blood gases and percentage saturation of hemoglobin with oxygen (Sat Hb). Arterial blood gases were measured using an ABL5 blood gas analyzer (Radiometer; Copenhagen, Denmark; measurements corrected to 38°C) (Fletcher et al. 2000; Thakor et al. 2010). Values for Sat Hb were determined using a hemoximeter (OSM3; Radiometer). Additional samples of maternal descending aortic blood (2 mL) were taken at set intervals during baseline and the experimental period for measurement of plasma hormone concentrations.

### Postmortem

At 138 days of gestation, pregant ewes were transferred from the hypoxic chambers to the postmortem laboratory wearing a respiratory hood providing the same hypoxic mixture. An additonal maternal blood sample (5 mL) was taken for future endocrine measurements. Under hypoxic conditions, ewes and their fetuses were killed by overdose of sodium pentobarbitone (0.4 mL kg^−1^ i.v. Pentoject; Animal Ltd., York, UK) and the fetus exteriorized by Cesarean section. A fetal umbilical artery blood sample (2 mL) was taken for measurement of fetal plasma cortisol levels. Heparinized saline (50,000 I.U) was then administered to the fetus via the umbilical vein, the fetus was weighed, and the heart was isolated and mounted in a Langendorff preperation. Fetal crown rump length and hind limb lengths were then determined. The upper hind limb comprised the length of the femur, the middle hind limb ranged between the patella and *tuber calcis*, and the lower hind limb ranged between the *tuber calcis* and the tips of the *phalanges*. One of the fetal hind limbs was then isolated for the myography experiments. Placentomes were classified into four categories by their gross morphological appearance, according to Vatnick et al. (1991). Following classification, the individual types were counted and weighed.

### Plasma hormone measurements

#### Catecholamines

Plasma adrenaline and noradrenaline were measured by a commercially available catecholamine ELISA kit (Abnova, Germany, Cat No. KA1877) according to the manufacturer's instructions. Two standard curves were plotted using a four‐parameter logistic fit in GraphPad (GraphPad Prism 6, GraphPad Software, Inc., CA) from which the adrenaline and noradrenaline concentrations of the unknown samples were determined. For adrenaline, the inter‐ and intra‐assay coefficients of variation were 15.6% and 12.6%, respectively, and the lower limit of detection was 0.01 ng mL^−1^. For noradrenaline, the inter‐ and intra‐assay coefficients of variation were 11.8% and 13.0%, respectively, and the lower limit of detection was 0.05 ng mL^−1^. The adrenaline and noradrenaline antisera had less than 0.2% cross‐reactivity for noradrenaline and adrenaline, respectively.

#### Cortisol

Plasma cortisol concentrations were measured using a commercially available ELISA kit (IBL international, Germany, Cat No. RE52061) according to the manufacturer's instructions. A standard curve was plotted using a four‐parameter logistic fit in GraphPad (GraphPad Prism 6) and from this the cortisol concentration in each sample was determined directly. For samples containing 43.1 and 223 ng mL^−1^ cortisol, the intra‐assay coefficients of variation were 2.90% and 2.57%, respectively. For samples containing 68.6 and 335 ng mL^−1^ cortisol, the interassay coefficients of variation were 5.00% and 2.13%, respectively. The lower limit of detection of the assay was 2.46 ng mL^−1^. The cross‐reactivity of the antiserum with other cortisol‐related compounds was 4.2% cortisone, 1.4% corticosterone, 0.4% progesterone, and 7.0% deoxycortisol.

### Langendorff preparation

The excised fetal heart was placed in ice‐cold Krebs–Henseleit bicarbonate buffer. It was then cannulated via the aorta (<2 min from excision) and perfused through the coronary arteries at a constant pressure of 30 mmHg, as detailed by Fletcher et al. (2005)). A pulmonary arteriotomy was also performed. A recirculating solution of Krebs–Henseleit bicarbonate buffer containing (mmol L^−1^) 120 NaCl, 4.7 KCl, 1.2 MgSO_2_.7H_2_O, 1.2 KH_2_PO_4_, 25 NaHCO_3_, 10 glucose, and 1.3 CaCl_2_.2H_2_O was filtered through a 5‐*μ*m cellulose nitrate filter (Millipore, Bedford, MA) and gassed with O_2_:CO_2_ (95:5) at 37°C. A small nonelastic balloon was inserted into the left ventricle through the left atrium. The balloon was filled with deionized water and attached to a rigid deionized water‐filled catheter connected to a calibrated pressure transducer (Argon Medical Devices, Athens, TX). The balloon volume was adjusted to obtain a left ventricular end diastolic pressure (LVEDP) recording of approximately 5–10 mmHg. After an initial 15‐min stabilization period, basal heart rate (HR), left ventricular systolic pressure (LVSP), and LVEDP were recorded. Basal left ventricular developed pressure (LVDP) was calculated as LVSP − LVEDP. The maximum and minimum first derivatives of the left ventricular pressure (dP/d*t*_max_ and dP/d*t*_min_) were calculated using an M‐PAQ data acquisition system (Maastricht Programmable AcQuisition System, the Netherlands).

### In vitro wire myography

Third order femoral arteries (internal diameter <300 *μ*m) were isolated and placed in physiological buffer solution (PBS). A segment of approximately 2 mm in length was cut and threaded with two 40 *μ*m diameter stainless steel wires. The vessel segment was then mounted on a wire myograph (Multi Wire Myograph System 610M; DMT, Denmark), while bathed in Krebs solution (mmol/L: NaCl 118.5, KCl 4.75, MgSO_4_ 7, H_2_O 1.2, KH_2_PO_4_ 1.2, NaHCO_3_ 25.0, CaCl_2_ 2.5, and glucose 5.5; Sigma) and constantly exposed to a gas mixture of 5% CO_2_ and 95% O_2_ at 37°C in the myograph chamber.

Following a 30‐min equilibration period, the vessel was stretched in a stepwise manner to a standardized tension equivalent to 40 mmHg physiological transmural pressure. Following a 20‐min equilibration period, a concentration–response curve to potassium (K^+^, 0–125 mmol/L) was generated to test maximal contractile capacity, and alpha‐adrenergic receptor reactivity to noradrenaline was also determined (NA; 10^−9^ to 10^−5^ mol/L; Sigma Aldrich, Poole, UK). All vessels were then precontracted with phenylephrine (PE, submaximal) before assessing endothelium‐independent vasodilator responses to sodium nitroprusside (SNP; 10^−9^ to 10^−4^; Sigma Aldrich) and endothelium‐dependent vasodilator responses to methacholine (MetCh; 10^−9^ to 10^−4^ mol/L; Sigma Aldrich). To determine the relative contribution of endogenous nitric oxide (NO), endothelium‐derived hyperpolarizing factor (EDHF) and prostanoid to endothelium‐dependent relaxation, concentration–response curves to MetCh were also generated following incubation for 10 min with l‐nitro‐arginine methyl ester (l‐NAME; 10^−5^ mol/L; Sigma Aldrich) and l‐NAME plus indomethacin (10^−6^ mol/L; Sigma Aldrich). Between experiments, vessels were washed repeatedly with Krebs solution and allowed to equilibrate for at least 20 min between different concentration–response curves.

Femoral arterial responses in the myography experiments were analyzed using Prism (v.5.0; GraphPad software). Concentration–response curves to K^+^ were analyzed using a Boltzmann sigmoidal curve, while all other curves were analyzed using a sigmoidal fit curve. The maximal effective tension (*E*_max_) and maximal constrictor response, expressed as a percentage of the contraction induced by 64.86 mmol/L K^+^ (%*K*_max_), were determined. The maximal relaxant response (%*R*_max_) was expressed as percentage of the contraction induced by PE and the vascular sensitivity was expressed as pD2 (pD2 = −log_10_ EC_50_, where EC_50_ is the dose required for 50% of the maximal effect). The contribution of NO‐dependent and ‐independent mechanisms of relaxation induced by MetCh was determined according to the methods of Herrera et al. (2010). The contribution of NO‐dependent mechanisms to the relaxation induced by MetCh was calculated by subtracting the area under the curve (AUC) for MetCh − the AUC for MetCh + l‐NAME. The contribution of NO‐independent mechanisms was the AUC for MetCh + l‐NAME. The contribution of prostanoid to the relaxation induced by MetCh was calculated as the AUC for MetCh + l‐NAME − the AUC for MetCh + l‐NAME + indomethacin. The remaining AUC following MetCh + l‐NAME + indomethacin was taken as EDHF.

### Measurement of arterial oxygenation in the fetus

To determine the effect of maternal exposure to this level of hypoxia on the fetal arterial oxygenation, a second cohort of animals (N, *n* = 6; H, *n* = 6) was similarly instrumented at 116 ± 1 days of gestation with maternal catheters under general anesthesia but had in addition a vascular catheter inserted into the fetal femoral artery. Pregnancies assigned to the chronic hypoxia group were then placed inside the chambers under normoxic conditions (11 L sec^−1^ air, equating to 39.6 m^3^ h^−1^) for 2 days. On the fifth postoperative day at 121 ± 1 days of gestation, pregnancies were exposed to 10% chronic hypoxia by altering the incoming gas mixture to 5 L sec^−1^ air: 6 L sec^−1^ N_2_ for 10 days. Maternal and fetal arterial blood samples (0.3 mL) were obtained every day for 10 days for measurement of arterial blood gases and SatHb, as before. Fetal measurements of arterial po_2_ were corrected to 39.5°C (Fletcher et al. 2000; Thakor et al. 2010; ABL5 blood gas analyzer, Radiometer; Copenhagen, Denmark).

### Statistical analysis

Data are expressed as mean ± SEM. Data were compared statistically either via the Student's *t*‐test for unpaired data or with a two‐way repeated measures ANOVA with the post hoc Tukey test, where appropriate. For all statistical comparisons, *P *<* *0.05 was considered statistically significant.

## Results

**Figure 2:**
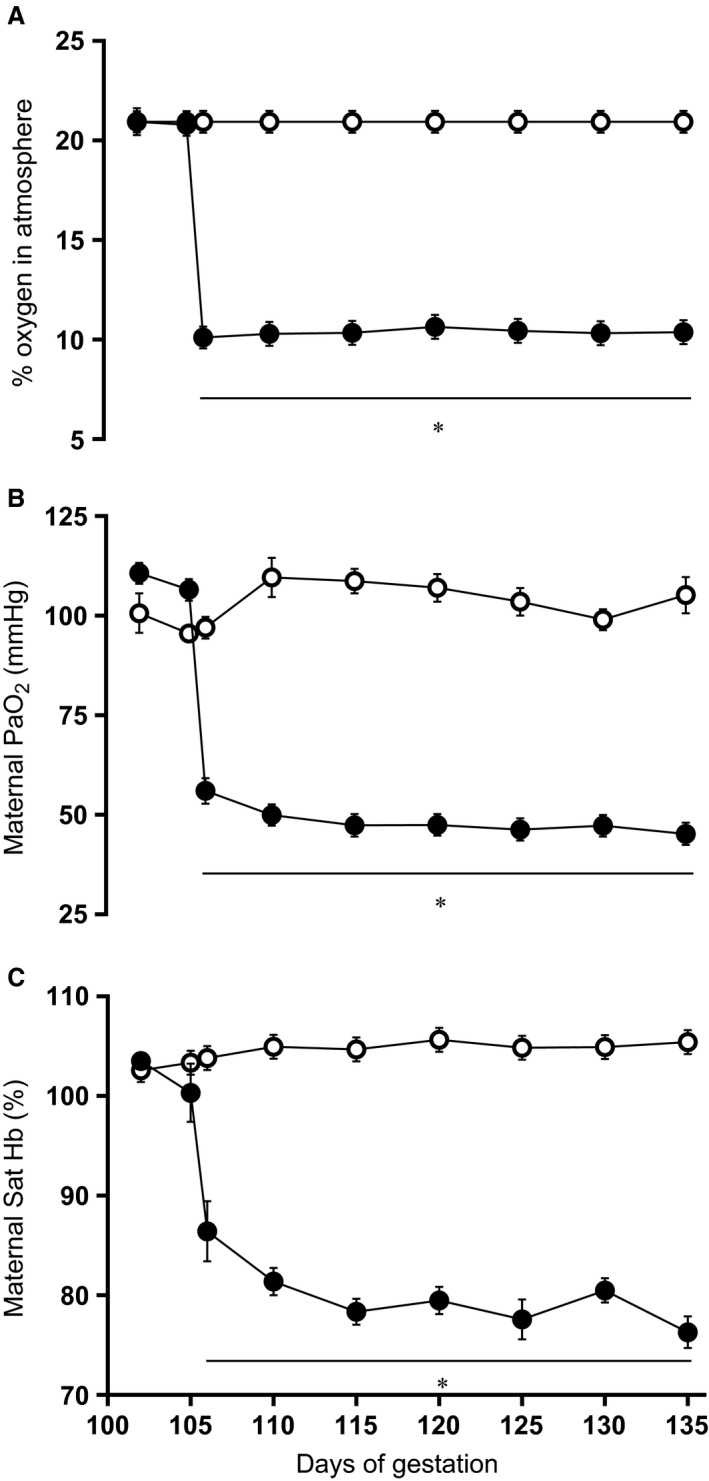
Maternal oxygenation. Values are mean ± SEM for the maternal atmospheric oxygen exposure (A), maternal arterial blood partial pressure of oxygen (PaO_2_; B), and arterial blood percentage saturation of hemoglobin with oxygen (Sat Hb; C). Groups are normoxia (N, ○, *n* = 10) and hypoxia (H, ●, *n* = 8). Significant (*P* < 0.05) differences are: * versus normoxia, two‐way repeated measures ANOVA with post hoc Tukey test.

**Figure 3:**
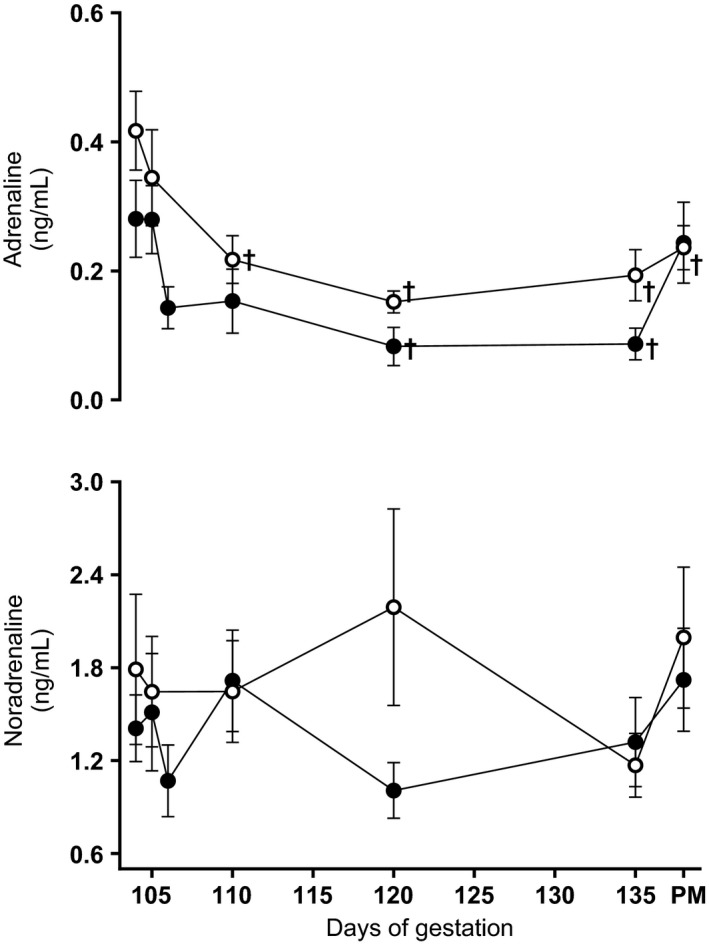
Maternal plasma catecholamine concentrations. Values are mean ± S.E.M. for the maternal plasma adrenaline and noradrenaline concentrations during the experimental period and at post mortem (PM). Groups are normoxia (N, ○, *n* = 9) and hypoxia (H, ●, *n* = 10). Significant (*P* < 0.05) differences are: †vs. baseline, Two‐Way repeated‐measures ANOVA with post‐hoc Tukey test.

**Figure 4:**
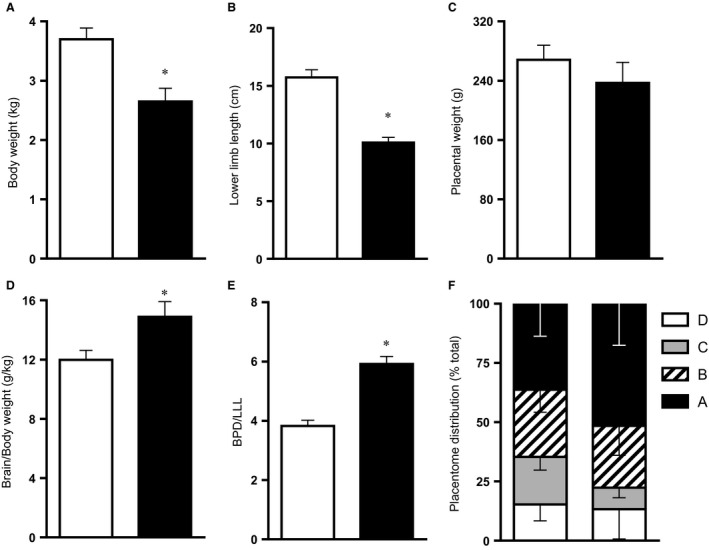
Fetal and placental morphometry. Values are mean ± SEM for the fetal body weight (A), lower limb length (B), placental weight (C), relative brain weight (D), biparietal diameter (BPD) relative to lower limb length (LLL; E), and placentome distribution (F) at 138 days of gestation. Groups are normoxia (N, □, *n* = 10) and hypoxia (H, ■, *n* = 8 for a, *n* = 6 for B–F). Hind limbs were divided into the following anatomical regions: The upper hind limb is the length of the femur, the middle hind limb ranging between the patella and tuber calcis, and the lower hind limb ranging between the tuber calcis and the tips of the phalanges. Significant (*P *<* *0.05) differences are: * versus normoxia, Student's *t*‐test for unpaired data.

**Figure 5:**
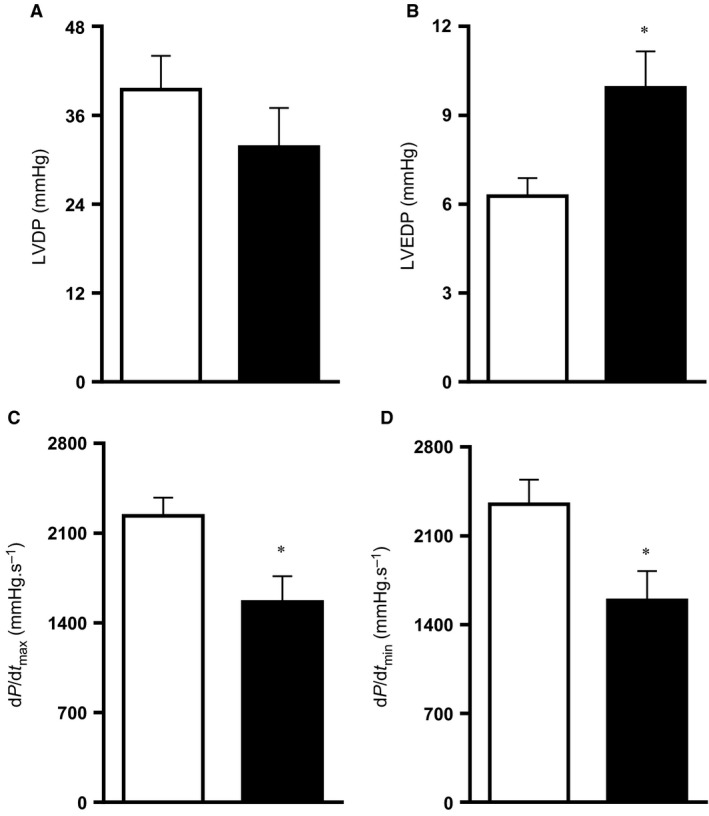
Fetal cardiac function. Values are mean ± SEM for the fetal heart left ventricular developed pressure (LVDP; A), left ventricular end diastolic pressure (LVEDP; B), the peak positive first derivative of left ventricular pressure (dP/d*t*_max_; C), and the peak negative first derivative of left ventricular pressure (dP/d*t*_min_; D). Groups are normoxia (N, □, *n* = 8) and hypoxia (H, ■, *n* = 8). Significant (*P* < 0.05) differences are: * versus normoxia, Student's *t*‐test for unpaired data.

**Figure 6:**
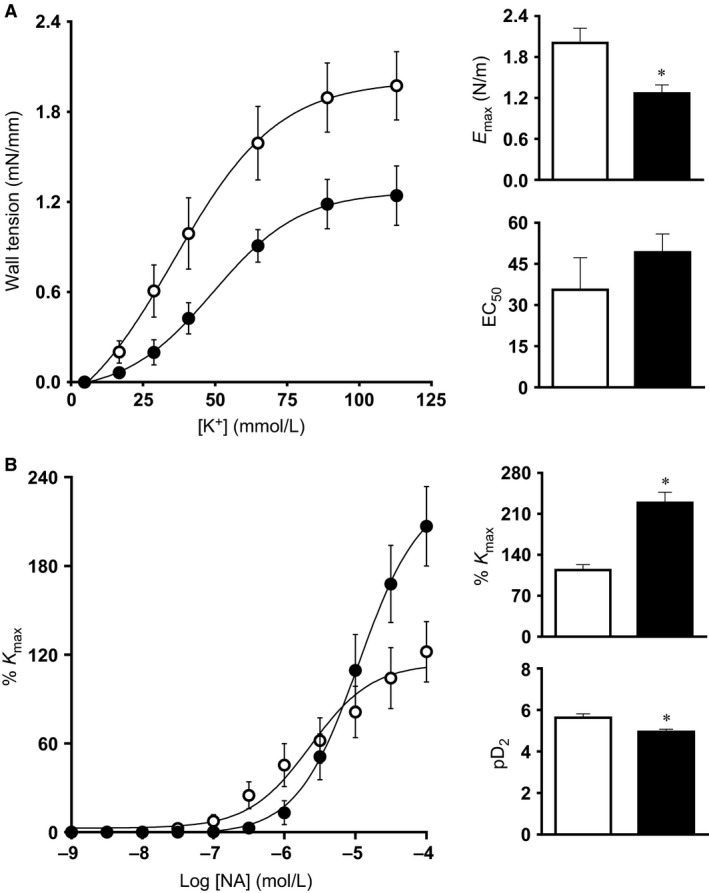
Fetal peripheral vasoconstrictor function. Values are mean ± SEM for the concentration–response curves, the maximal constriction (*E*_max_ or %*K*_max_), and the sensitivity (EC50 or pD2) to potassium (K+; A) and to noradrenaline (NA; B). Groups are normoxia (N, ○, *n* = 10) and hypoxia (H, ●, *n* = 8). Significant (*P* < 0.05) differences are: * versus normoxia, Student's *t*‐test for unpaired data.

**Figure 7:**
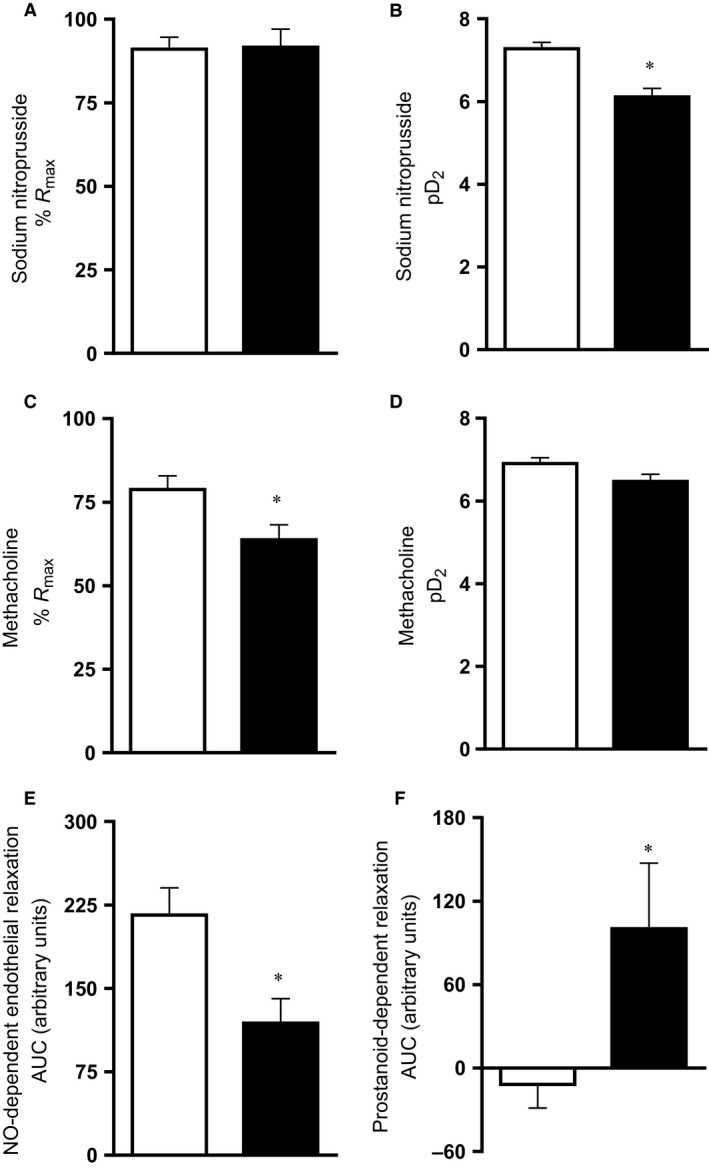
Fetal peripheral vasodilator function. Values are mean ± SEM for the maximal relaxation (%*R*_max_), and the sensitivity (pD2) to sodium nitroprusside (SNP; A and B) and to methacholine (MetCh; C and D). The nitric oxide (NO)‐dependent component to the endothelial‐dependent femoral vasorelaxation is shown in (E). The prostanoid‐dependent component to the NO‐independent femoral vasorelaxation is shown in (F). AUC, area under the curve. Groups are normoxia (N, □, *n* = 10) and hypoxia (H, ■, *n* = 8). Significant (*P* < 0.05) differences are: * versus normoxia, Student's *t*‐test for unpaired data.

Induction of significant hypoxia of 10% O_2_ to pregnant sheep over the last third of gestation induced controlled reductions in maternal Pao_2_ and hemoglobin oxygen saturation (Fig. 2) without affecting the maternal food intake. Basal maternal daily food consumption was not different between groups (N: 1.3 ± 0.9 vs. H: 1.1 ± 0.4 kg day^−1^). Exposure to chronic hypoxia did not affect maternal food intake (N: 1.5 ± 0.9 vs. H: 1.5 ± 0.9 kg day^−1^). Maternal basal plasma catecholamine levels did not differ between groups and concentrations of adrenaline and noradrenaline did not increase above baseline during chronic hypoxia (Fig. 3). In addition, fetal plasma cortisol levels measured at post mortem were not different between normoxic and chronic hypoxic pregnancy (N, *n* = 8: 17.6 ± 3.0 vs. H, *n* = 8: 16.6 ± 2.9 ng mL^−1^). This level of maternal hypoxia produced mean fetal descending arterial po_2_ values of 11.5 ± 0.6 mmHg relative to a mean 20.9 ± 0.5 mmHg in control fetuses of normoxic pregnancy, when fetal blood samples were monitored between 125 and 138 days of gestation (*P* < 0.05). Chronic hypoxia of this level during the last third of gestation led to reductions in fetal body weight and fetal lower limb length (Fig. 4A and B). In contrast, reductions in fetal brain weight were comparatively smaller, such that the ratio of the brain to body weight or of the biparietal diameter to lower limb length was significantly increased in hypoxic fetuses (Fig. 4D and E). Therefore, this model of experimental hypoxia in sheep reproduced the significant asymmetric fetal growth restriction, characteristic of hypoxic pregnancy associated with FGR in humans (Wollmann 1998). The ovine placenta is composed of units known as placentomes. These were classified into four categories by their gross morphological appearance, according to Vatnick et al. (1991). The placentome type distribution appeared to be altered toward a greater proportion of placentomes of the A type (51.6 ± 17.5% vs. 36.3 ± 13.7%) and a lower proportion of placentomes of the D type (13.3 ± 12.6% vs. 15.3 ± 6.9%) in placentas from chronic hypoxia relative to control pregnancy (Fig. 4F). However, this trend did not reach statistical significance and the comparison may reflect the possibility of Type II error. Hearts isolated from chronically hypoxic fetuses showed significantly elevated values for left ventricular end diastolic pressure (LVEDP) while having reduced myocardial contractility (dP/d*t*_max_) and relaxability (dP/d*t*_min_) relative to hearts isolated from normoxic fetuses (Fig. 5). Femoral resistance vessels isolated from chronically hypoxic fetuses showed significantly impaired constrictor responses to potassium with enhanced constrictor responses to noradrenaline (Fig. 6). Chronically hypoxic fetuses also showed reduced femoral vasodilator responses to sodium nitroprusside and to methacholine (Fig. 7). To further determine the relative contributions of endogenous nitric oxide (NO), endothelium‐derived hyperpolarizing factor (EDHF), and prostanoids to the endothelium‐dependent relaxation, concentration–response curves to methacholine were also generated following incubation with the NO synthase inhibitor l‐NAME, and following l‐NAME plus the nonselective inhibitor of cyclooxygenase, indomethacin. These additional experiments revealed that the impaired femoral endothelial‐dependent relaxant response in chronically hypoxic fetuses was NO dependent. However, femoral vessels of chronically hypoxic fetuses also showed a markedly enhanced prostanoid‐dependent relaxation compared with vessels from fetuses of control pregnancy (Fig. 7E and F).

## Discussion

Exposure of pregnant sheep to significant continuously monitored hypoxia during the last third of gestation in the present study reproduced hallmarks of the impaired fetal cardiac phenotype previously described in other models of compromised development as well as in the human FGR fetus. Elegant studies by the laboratories of Gilbert, Longo, and Pearce reported a decrease in cardiac output in the chronically hypoxic sheep fetus secondary to a decrease in myocardial cell contractile function (see Gilbert 1998). Involved intracellular mechanisms included reduced myofibrillar Mg(^2+^)‐activated ATPase and a decrease in *β*_1_‐adrenoreceptor influence on cardiac contraction. Similarly, in the chick embryo, the laboratory of Keller and colleagues (Sharma et al. 2006) reported that chronic hypoxia led to impaired ventricular +dP/d*t* and decreased ventricular ejection fraction. Four human clinical studies have now reported that babies born from pregnancies complicated by placental insufficiency show cardiac compromise. Abnormalities in cardiac morphology and function of the human FGR infant also included compromised biventricular ejection force (Rizzo et al. 1995) with significant diastolic dysfunction (Miyague et al. 1997), ventricular wall hypertrophy (Veille et al. 1993), and a decrease in ventricle and myocyte volume (Mayhew et al. 1999).

An inverse relationship between birth weight, a constrictor phenotype, and endothelial dysfunction in the peripheral vasculature in early life is well described, even in humans (Leeson et al. 1997, 2001). Previous studies in the chick embryo and in fetal sheep have reported that chronic hypoxia during development promotes an enhanced vasoconstrictor phenotype in peripheral resistance circulations, which is mediated by increased sympathetic influences coupled with loss of NO‐dependent endothelial function. For instance, the enhanced vasoconstrictor reactivity to noradrenaline in isolated femoral vessels from chronically hypoxic fetal sheep reported in the present study is consistent with reports of sympathetic hyperinnervation of peripheral resistance arteries by the end of the incubation in the chronically hypoxic chick embryo (Rouwet et al. 2002) as well as an increased sensitivity to *α*_1_‐adrenergic receptor agonists in the peripheral circulation of chronically hypoxic sheep (Kim et al. 2005).

Similarly, several studies have reported consistent loss of endothelial function in offspring of chronically hypoxic pregnancy mediated largely via an impairment of NO‐dependent dilator mechanisms (see Giussani and Davidge 2013; Giussani 2015). Fewer studies have investigated whether endothelium‐dependent pathways other than NO are affected by growth restriction in utero. An interesting study by Morton et al. (2010) reported maintained overall vasodilator responses in rat offspring of hypoxic pregnancy despite impaired NO‐dependent relaxation in mesenteric arteries. These findings highlighted the possibility of compensatory dilator mechanisms which are recruited to maintain peripheral dilator capacity upon loss of NO‐dependent endothelial function. In the present study, the significantly enhanced prostanoid‐dependent relaxation despite impaired NO‐dependent endothelial function in femoral arteries isolated from chronically hypoxic fetuses may be an example of similar compensation by alternative vasodilator mechanisms.

It is well established that in response to acute hypoxia, the late gestation fetus redistributes its cardiac output away from peripheral circulations to spare the brain (Rudolph 1984; Giussani et al. 1994; Giussani 2015). While beneficial to the developing central nervous system, persistent redistribution of blood flow away from less essential vascular beds during chronic fetal hypoxia triggers unwanted adverse side effects. Among these is asymmetric FGR, characterized by babies having an appropriate head size with a shorter body length or by babies being thin for their length, with a reduced ponderal index (Barker 1994). Exposure of pregnant sheep to significant hypoxia during the last third of gestation in the present study reproduced this fetal body phenotype, characteristic of human FGR pregnancy. This is important because Barker et al. (1989) reported that thinness at birth was the variable best associated with increased rates of future cardiovascular disease in human populations.

Adverse intrauterine conditions early in gestation have previously been shown to lead to a shift from A‐ to D‐type placentomes later in gestation (Penninga and Longo 1998; Heasman et al. 1999; Steyn et al. 2001). For instance, an increase in the number of D‐type placentomes has been observed in response to maternal undernutrition (Steyn et al. 2001), carunclectomy (Robinson et al. 1979), and high‐altitude chronic hypoxia (Penninga and Longo 1998) in sheep. Consequently, early ideas on shifts in placentome morphology suggested that the presence of more C‐ and D‐type placentomes was an adaptation to increase nutrient delivery to the compromised fetus (Alexander 1964; Robinson et al. 1997; Bell et al. 1999). However, several more recent studies have now confirmed that there is in fact no effect on placental nutrient transfer to the fetus (Ward et al. 2006) or on placental vascularity (Vonnahme et al. 2008) of alterations in placentome morphology in sheep. In a study by Gardner et al. (2002) it was found that short‐term umbilical cord occlusion decreased the number of C‐ and D‐type placentomes, which suggested a reduction in umblical blood flow may be responsible for a shift toward A‐ and B‐type placentomes. Other studies have suggested that cortisol exposure may be a defining factor, as fetal cortisol infusion prevented the normal ontogenic shift in placentome morphology from A‐ to D‐types, resulting in a greater proportion of A‐ and B‐type placentomes (Ward et al. 2006). In the present study, the shift toward a greater number of A‐type placentomes in chronically hypoxic pregnancies resembles the effect on placentome morphology of prolonged umbilical cord compression (Gardner et al. 2002) but is discordant with the effects of high‐altitude pregnancy (Penninga and Longo 1998) in sheep. These results on placentome morphology are also unlikely to be attributable to alterations in glucocorticoid concentrations (Ward et al. 2006) as fetal plasma cortisol levels were not different between normoxic and hypoxic pregnancy. The mechanisms that control the proportions of different placentome types in this and other studies in sheep therefore remain unknown.

In conclusion, the work introduces to the field of study a new technique for physiological research able to measure in chronically instrumented maternal and fetal sheep significant and controlled chronic fetal hypoxia beyond levels that can be achieved by habitable high altitude. This level of chronic hypoxia induces significant asymmetric fetal growth restriction, akin to that observed in human FGR pregnancy. An additional advantage of the technique is that this level of hypoxia in sheep does not affect maternal food intake or maternal or fetal stress hormones. This level of chronic fetal hypoxia leads to a fetal origin of cardiovascular dysfunction, characterized by impaired myocardial contractility and diastolic function as well as alterations in constrictor reactivity and loss of NO‐dependent endothelial function in peripheral resistance circulations. These cardiovascular deficits are similar to those described in human FGR pregnancy. Therefore, the technique introduced to the field of study is well poised to isolate the contribution of chronic fetal hypoxia to developmental programming of cardiovascular disease in a large mammalian species, such as the sheep, which not only permits chronic instrumentation of the fetus but also has a prenatal temporal profile of cardiovascular development that closely resembles that in humans.

## Conflict of Interest

None declared.
